# Muscle fatigue in relation to forearm pain and tenderness among professional computer users

**DOI:** 10.1186/1745-6673-2-17

**Published:** 2007-12-08

**Authors:** Gert F Thomsen, Pete W Johnson, Susanne W Svendsen, Ann I Kryger, Jens Peter E Bonde

**Affiliations:** 1 Department of Occupational Medicine, Aarhus University Hospital, Noerrebrogade 44, building 2 C, 8000 Århus, Denmark; 2 Department of Occupational Medicine, Ribe County Hospital, Oestergade 80, 6700 Esbjerg, Denmark; 3 Department of Environmental and Occupational Sciences, School of Public Health, Seattle, USA; 4 Department of Occupational and Environmental Medicine, Copenhagen University Hospital, Bispebjerg Bakke 23, 2400 Copenhagen NV, Denmark

## Abstract

**Background:**

To examine the hypothesis that forearm pain with palpation tenderness in computer users is associated with increased extensor muscle fatigue.

**Methods:**

Eighteen persons with pain and moderate to severe palpation tenderness in the extensor muscle group of the right forearm and twenty gender and age matched referents without such complaints were enrolled from the Danish NUDATA study of neck and upper extremity disorders among technical assistants and machine technicians. Fatigue of the right forearm extensor muscles was assessed by muscle twitch forces in response to low frequency (2 Hz) percutaneous electrical stimulation. Twitch forces were measured before, immediately after and 15 minutes into recovery of an extensor isometric wrist extension for ten minutes at 15 % Maximal Voluntary Contraction (MVC).

**Results:**

The average MVC wrist extension force and baseline stimulated twitch forces were equal in the case and the referent group. After the fatiguing contraction, a decrease in muscle average twitch force was seen in both groups, but the decrease was largest in the referent group: 27% (95% CI 17–37) versus 9% (95% CI -2 to 20). This difference in twitch force response was not explained by differences in the MVC or body mass index.

**Conclusion:**

Computer users with forearm pain and moderate to severe palpation tenderness had diminished forearm extensor muscle fatigue response. Additional studies are necessary to determine whether this result reflects an adaptive response to exposure without any pathophysiological significance, or represents a part of a causal pathway leading to pain.

## Introduction

Intensive use of mouse and keyboard among professional computer users has been identified as a risk factor for pain in various regions of the upper extremity including the forearm [1-3]. The mechanism and pathophysiology of the pain response are not well understood [4]. In most studies pain complaints are poorly associated with commonly accepted criteria for specific clinical diagnoses.

Muscle fatigue can be defined as an exercise induced transient decrease in the force generating capacity of the muscle [5]. While electromyography (EMG) can be used to measure muscle fatigue in moderate to high force work [6,6], it is rather insensitive to fatigue developed during the performance of low force occupational activities such as the use of mouse and keyboard [7,8]. The ratio of force output from low and high frequency (e.g. 20 Hz and 100 Hz) electrical stimulation has been used as a measure of muscle fatigue [9-11]. In some persons high frequency stimulation is very unpleasant, which makes this method less suitable for epidemiological studies and which may introduce selection problems. In 1998 Johnson demonstrated that the a muscle's force (twitch) response following very low frequency (2 Hz) electrical stimulation of a forearm flexor muscle was a reliable method to measure muscle fatigue [12,13]. Significant but transient levels of muscle fatigue were observed in computer users who applied average forces between 0.7 and 6.5% of the maximal voluntary contraction (MVC) for 3 to 4 hours [12]. However, EMG measurements indicate higher load of the forearm extensor muscle groups compared to the flexor muscle groups during use of the mouse and keyboard [14-17]. Therefore we adapted the 2 Hz stimulation technique used by Johnson and others [13,18] to measure muscle fatigue of the forearm extensor muscles.

The purpose of the study was to investigate the hypothesis that computer users with forearm pain have a higher level of extensor muscle fatigue than computer users without forearm pain [17]. The study is part of the Danish nationwide NUDATA study of neck and upper extremity disorders among technical assistants and machine technicians [2].

## Subjects and methods

### Selection of participants and assessment of forearm pain and tenderness

Computer users with forearm pain (cases) and without forearm pain (referents) were recruited among respondents in the NUDATA study. In January through June 2000 6,943 participants of 9,480 (73%) technical assistants and machine technicians completed a baseline questionnaire on job tasks, lifestyle and pain in the upper extremities including the forearms [2]. Forearm pain within the past seven days was assessed on a nominal scale with eight pain categories (no pain, very little pain, little pain, little to moderate pain, moderate pain, moderate to severe pain, severe pain, and very severe pain). Subjects reporting at least moderate pain in one or both forearms during the past 7 days were defined as symptom cases in the NUDATA study and were offered a clinical examination that took place within 14 days of receipt of the questionnaire data. Palpation tenderness in the proximal lateral aspect of the forearm extensor muscle group was assessed using a digital pressure of approximately 4 kg perpendicular to the surface. The response was scored on a 0–3 scale (0, none; 1, mild without withdrawal; 2, moderate with withdrawal; 3, severe with jump sign).

### Eligibility criteria

#### Assistants and technicians with forearm pain (cases)

Among participants who reported at least moderate pain in the right forearm during the past 7 seven days and at the clinical examination showed moderate or severe palpation tenderness, we invited the first 24 consecutive cases in the Aarhus region to take part in this study. Twenty persons accepted, but two declined participation after enrolment. Specific job tasks as work at video display terminals were not requested but due to the sampling frame all participants used computers to some degree in their daily work.

#### Assistants and technicians without forearm pain (referents)

For each accepting case we enrolled concomitantly from the same cohort and geographic area a participant of same sex and age (within a 5 year interval) without any self-reported pain in the upper extremities during the last 12 months. The referents that matched the two case subjects who withdrew after enrolment were kept in the study. None of the referents had become cases during the time elapsed from filling in the questionnaire and enrolment into this study.

#### Exclusion criteria

Assistants and technicians with a history of surgery involving the right extremity, trauma sequelae, epicondylitis, carpal tunnel syndrome and arthritis were not eligible for the study. Furthermore, workers with abnormal range of movement in the right shoulder, elbow, wrist or fingers or with signs of acute inflammation (swelling, rubor and increased skin temperature) in these regions were excluded.

**Figure 1:**
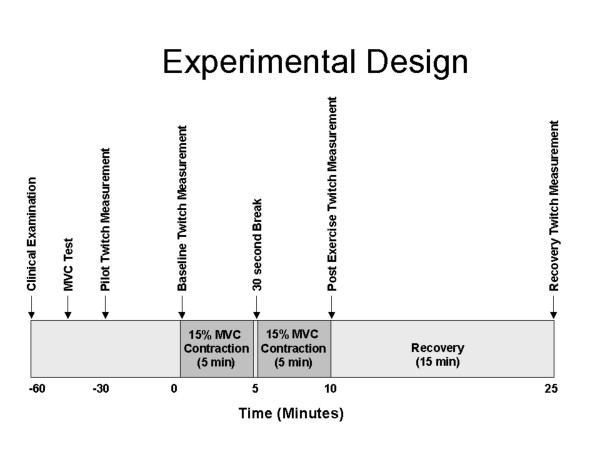
Experimental design and protocol for measurements of right forearm extensor muscle twitch forces in 38 whitecollar workers with and without forearm pain.

All examinations were performed at the Department of Occupational Medicine αt Aarhus University Hospital. Cases and controls were intermingled across time and the examiner was blinded to case or control status. The time schedule for the tests for one subject can be seen in Figure 1. The regional ethical committee approved the study and all subjects signed an informed consent prior to enrolment into the present study.

### MVC-measurements

**Figure 2:**
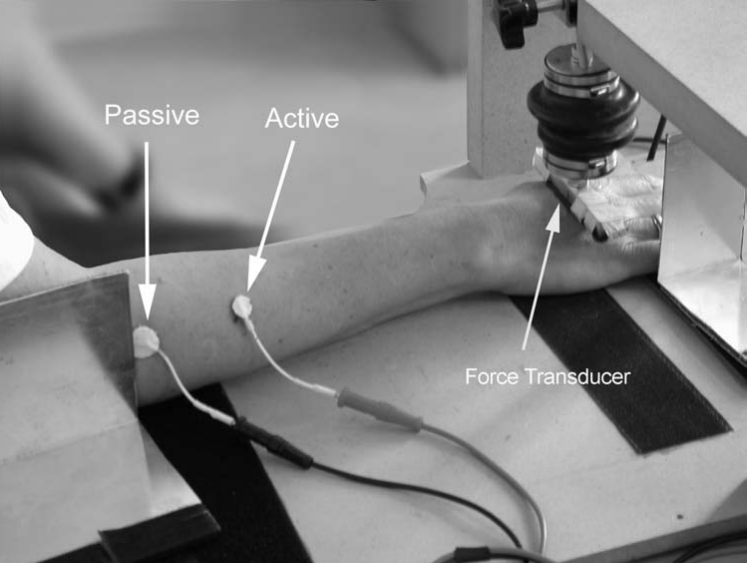
Placement of electrodes and force transducer in a study of right forearm extensor muscle twitch forces. A vertical plate to stabilize the distal forearm and wrist region sideways is not shown in order not to hide the transducer.

The maximal voluntary wrist extension forces were measured using a force measurement apparatus with a standard voltmeter reading the amplifier output voltage. The measurements were calibrated using laboratory grade weights. Each subject performed three maximum exertions while encouraged to extend the wrist and fingers as forceful as possible. The posture of the forearm is indicated in Figure 2. Participants were allowed to relax for a couple of minutes between each exertion. The highest recorded of the three force readings defined the subject's MVC.

### Twitch force measurements

Muscle twitches were evoked using a custom built timer and a Digitimer DS7A Constant Current Electrical Stimulation Unit. Figure 2 shows the experimental set-up. The right forearm extensor muscles were stimulated using a 12 mm (active) Stimtrode Ag/AgCl electrode, which was placed on the proximal lateral aspect of the forearm one third of the distance between the elbow and wrist. The electrode was placed over the muscle belly that could be felt/palpitated when the third finger was extended. A 20 mm (passive) electrode was placed just anterior-medial to caput radii at the elbow. The muscle was stimulated with 100 microsecond square pulses at a frequency of 2 Hz. The twitch forces were measured with an Omega LC105 force transducer and an Omega DMD 465 amplifier (Omega Engineering Inc.; Stamford, CT USA) placed as indicated in Figure 2. In setting up the experimental procedures it proved more convenient to measure extensor muscle twitch force at the metacarpophalangeal (MCP) joint rather than a position distal to this location. Data was collected with an IBM PC instrumented with a data acquisition card (model AT-MIO-16E; National Instruments; Austin, TX USA) running Labview Software (version 3.0; National Instruments; Austin, TX USA). The sampling frequency was 1000 Hz.

### Calibration of the stimulation current

After the hand had been placed in the apparatus (Figure 2), the current was gradually increased up to or above 40 mA. In a pilot study, this current level was tolerable to most persons and produced contractions with sufficient force (> 1.0 N) to ensure reliable measurements. Nine persons did not tolerate 40 mA, but currents ranging between 32.5 and 37.5 mA produced reproducible twitch responses. In other seven persons 40 mA produced contractions that were insufficient to ensure reliable force measurements. In these subjects the current was increased to 42 – 50 mA. For each individual, the established current level was kept through the rest of the study. To accustom the subject to the procedures, a full pilot measurement was performed 30 minutes prior to the experiment.

### Experimental procedure

In the experiment the subject performed an exercise consisting of a static wrist extension at 15% of MVC for 5 minutes, 30 seconds break and another 5 minutes 15% MVC wrist extension. The duration of the exercises was set according to earlier experience [19,20]. The force was measured in the same way as at the MVC-manoeuvre. The subject could see the force reading and was urged by the researcher to keep the force level. Before (baseline), immediately after the exercise (post-exercise), and 15 minutes after exercise (recovery) twitch measurements were collected.

**Figure 3:**
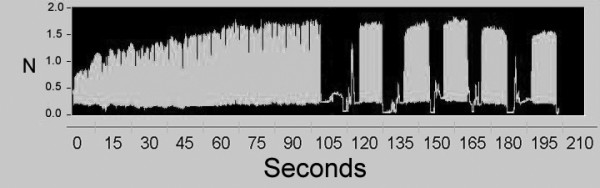
Time-force record (screen printout) from a single measurement consisting of 90 seconds of conditioning followed by five trains of approximately 30 twitch stimulations separated by removal and repositioning of the hand.

It is well known that the muscle force increases during the initial phase of a muscle contraction – whether stimulated or voluntary [21,22]. At each measurement (baseline, post-exercise, and recovery), the subject's muscle was therefore first conditioned with 90 seconds continuous 2 Hz electrical stimulation in order to reach the plateau of steady state twitch force [12,13,18]. Immediately after the conditioning, muscle twitch forces were measured during five trains of approximately 30 twitch stimulations. Between each of the five trains, subjects removed and repositioned their hands in the measurement apparatus in order to minimize minor effects that hand position might have on recorded twitch force (Figure 3). The hand repositioning between trains typically took less than five seconds.

### Statistical analyses

For the five trains collected from each subject at each time period, the between-5 train coefficient of variation (CV) was computed by dividing the train standard deviation by the mean [23]. Average values were then computed across times for the cases and referents, respectively.

To allow comparisons of twitch measurements between subjects, twitch force data were standardized with respect to the baseline measurements performed before the voluntary wrist extension exercise. A repeated measures analysis of variance [SAS version 8.02 Proc Mixed random (SAS Institute, Cary, NC)] was used to determine whether the twitch forces differed after the exercise in comparison with baseline levels among cases and referents and whether the changes relative to baseline values differed between the two groups. In all analyses, the dependent variable was the standardized twitch force of the single contractions and independent variables included case status (case/referent) and time (baseline, post-exercise, recovery). In additional analyses the possible confounding effects of a number of extraneous factors were evaluated in multiple regression analyses. These factors included gender, age (> = 40 years: yes/no), current smoking (yes/no), body mass index (BMI) (> 27 kg/m^2^: yes/no) and physical activity in leisure time (three levels).

## Results

The characteristics of the study population are outlined in Table 1. The majority of participants were women (over 80%) and the average age was 43.4 years (range 25–55 years). The distributions of lifestyle factors were similar in the case and the reference group but the cases reported slightly more working hours, computer work and mouse use. The average force recorded during maximal voluntary extension of the wrist was at the same level among cases and referents. The baseline stimulated twitch forces averaged 2.29 (SD 1.58 N) showing no difference between the case group and the reference group. The coefficients of variation in the three series of measurements are displayed in Table 2. The reliability of the twitch force measurement was very high – on average, the coefficient of variation between 5 trains was 1.2 %.

**Table 1:** Characteristics of study groups

**Characteristics**	**Cases**	**Referents**
Gender, n (%)		
Female	15 (83%)	17 (85%)
Male	3 (17%)	3 (15%)
		
Age, years, mean (min-max)	43.0 (25.5–53.4)	43.8 (28.4–55.2)
Body mass index, kg/m^2^, mean (min-max)	24.6 (30.7–19.3)	23.6 (34.6–19.5)
Smoking, n (%)		
Never smoking	8 (44%)	12 (60%)
Ex-smoker	7 (39%)	4 (20%)
Current smoker	3 (17%)	4 (20%)
		
Leisure time physical activity, n (%)		
Less than 2 hours/week	5 (28%)	4 (20%)
2–4 hours/week	8 (44%)	10 (50%)
More than 4 hours/week	5 (28%)	6 (20%)
		
Working conditions^1^, mean (SD)		
Work hours/week	37.0 (5.0)	35.5 (3.3)
Computer use, hours/week	28.6 (5.7)	23.9 (9.3)
Mouse use, hours/week	22.0 (11.1)	18.9 (12.8)
		
Wrist extension force, mean (SD)		
Maximal voluntary contraction (Newton)	107.9 (51.5)	110.1 (32.6)
Stimulation current (mA), mean (SD)	40.1 (4.9)	39.6 (3.1)
Twitch force at baseline (Newton), mean (SD)	2.32 (1.63)	2.27 (1.56)

^1^Self reported, questionnaire

**Table 2:** Average and range of the between-5 train twitch force coefficient of variations collected at each time period and grouped by cases and referents.

**Measurement time period**	**Cases [n = 18] Coefficient of Variation, %, Mean (min-max)**	**Referents [n = 20] Coefficient of Variation, % Mean (min-max)**
Baseline	1.6 (0.1–13.4)	1.9 (0.2 – 14.7)
Post exercise	1.0 (0.1 – 3.9)	1.2 (0.1 – 8.4)
Recovery	0.5 (0.0 – 2.1)	1.0 (0.1 – 5.6)

Among the referents we observed extensor muscle fatigue following the 10 minutes exercise at 15% of MVC as indicated by a 17 % decline in twitch force compared to baseline levels. The fatigue developed further during the first 15 minutes of the recovery phase (Table 3).

**Table 3:** Average standardised forearm extensor muscle twitch forces among subjects with and without forearm pain and tenderness measured at baseline, after 10 minutes exertion at 15% MCV, and 15 minutes into recovery.

	Cases n = 18		Referents n = 20		
		
	Mean	95% CI	Mean	95% CI	P-value^1^
Baseline	1.00	0.99–1.01	1.00	0.99–1.01	0.937
Post exercise	1.01	0.89–1.12	0.83	0.72–0.94	0.032
Recovery	0.91	0.80–1.02	0.73	0.63–0.83	0.017

All analyses were adjusted for the train number (1–5) and multiple measurements (30 in each train).

^1 ^Test for difference of mean values in cases compared to referents.

Among the cases we did not observe any reduction in muscle force immediately after the exercise but a slight decrease in twitch force during the recovery period. Current smoking, age and MVC were not related to muscle fatigue but higher BMI was associated with a higher degree of fatigue (p = 0.01). Inclusion of these covariates into the models did not change the displayed effects of exercise on twitch forces.

## Discussion

In this study, computer users with pain and moderate to severe palpation tenderness in the forearm experienced less forearm extensor muscle fatigue after an exercise protocol than a healthy referent group. This finding is contrary to our *a- priory *hypothesis stating that pain and tenderness of the forearm would be associated with increased muscle fatigue.

The observed muscular fatigue response among the referents is consistent with earlier findings. Blangsted observed muscular fatigue as measured with mechanomyography and electromyography in the extensor carpi radialis muscle 30 minutes following a 10 minutes 10% MVC wrist extension [19]. In Johnson's experiments a 10% reduction in flexor muscle twitch force was observed immediately after 10 minutes static contractions at 15% of MVC, which increased to 20% reduction after 30 minutes [12]. Following a similar provocation of the forearm extensor muscles in our referent group we observed 17% reduction in twitch force immediately after the contraction, which increased to 27% after 15 minutes. In contrast, in our cases we saw virtually no change in twitch forces immediately after the contraction and only a 9% decrease 15 minutes into the recovery period. Referents and cases were investigated in random order and thus a change in measurement conditions or the experimental set-up across the study period is not likely to explain our findings. Moreover, the very low coefficient of variation of less than 5% in the majority of measurements indicates that our methods were reliable.

A diminished fatigue response among the persons with forearm pain might result if the pain caused the subjects to produce reduced force at the maximal voluntary contraction and subsequently cause reduced power at the exercise before the twitch measurements. However, the findings are not the result of a lower force output in persons with forearm pain since the absolute force during stimulated contractions, the contraction force during exercise and the maximum voluntary contractions were at the same level in the two groups.

Cases and referents were recruited among members of the same trade union and were therefore expected to be socially and economically rather homogeneous. Care was taken to ensure equal gender and age distributions in the two samples and cases and referents were similar with respect to a number of physical and lifestyle characteristics. A higher BMI was associated with increased fatigue but inclusion of this variable as well as leisure time physical activity and current tobacco smoking did not change the observed associations between study group and degree of fatigue. Nor was the level of statistical significance affected. One to five months had past from the assignment to the case and referent group until the measurements were made. However, on the day of the experiment the clinical examination assured that the referents had not developed any muscle tenderness. Any bias due to the cases becoming non-cases would lead to an underestimation of the true difference between the groups.

Accordingly, we do not believe that the findings can be explained by errors in the measurement technique or biased statistical comparisons but obviously there is a need to corroborate or refute the findings in independent studies. Assuming that the findings reflect genuine biological differences in muscle function among subjects with and without forearm pain we need new hypotheses to understand the results. Due to the cross-sectional study design we cannot infer whether the diminished fatigue response precedes the development of pain and maybe makes the muscle more vulnerable to exposure, or whether it is a correlate or consequence of pain.

It has been hypothesized that the patophysiology of upper extremity muscle disorders including forearm pain in computer users are caused by disorders of muscle cells or limitations of the local circulation [24-26]. The Cindarella hypothesis proposes that the development of chronic muscular pain is due to an overuse of fibers belonging to low-threshold motor units. It has in a study been demonstrated, indeed, that there are motor units that are continuously active under a 25 minutes static low-level excertion of the extensor digitorum communis muscle while the majority of motor units were only partially active over time [25]. Although speculative, it can be hypothesized that forearm pain develop more frequent in workers with a larger proportion of Cindarella fibers and if such fibers are less likely to be fatigued by exercise this could explain the limited decline in forearm muscle twitch among cases in out study. If so, we would expect to observe the same diminished fatigue pattern in the contra lateral forearm without pain. Unfortunately this was not measured in this study.

## Conclusion

Computer users with moderate to severe forearm pain had a diminished forearm extensor muscle fatigue response. It cannot be inferred from this study whether the abnormal fatigue pattern is a result of the pain or is part of the causal mechanisms leading to pain. The findings need to be corroborated and further explored in additional studies.

## Competing interests

The author(s) declare that they have no competing interests.

## Authors' contributions

SWS and JPB conceived the study. GT and PWJ designed the experimental set-up and performed all measurements and tests. GT and JPB analyzed the data and drafted the manuscript. All contributed to revisions and approved the final version of the paper.
